# Silicone Wristbands in Exposure Assessment: Analytical Considerations and Comparison with Other Approaches

**DOI:** 10.3390/ijerph19041935

**Published:** 2022-02-09

**Authors:** Małgorzata Wacławik, Wojciech Rodzaj, Bartosz Wielgomas

**Affiliations:** Department of Toxicology, Faculty of Pharmacy, Medical University of Gdańsk, 107 Hallera Street, 80-416 Gdańsk, Poland; malgorzata.waclawik@gumed.edu.pl (M.W.); wojciech.rodzaj@gumed.edu.pl (W.R.)

**Keywords:** biomonitoring, exposome, human exposure, silicone wristband, passive sampling, personal monitoring

## Abstract

Humans are exposed to numerous potentially harmful chemicals throughout their lifetime. Although many studies have addressed this issue, the data on chronic exposure is still lacking. Hence, there is a growing interest in methods and tools allowing to longitudinally track personal exposure to multiple chemicals via different routes. Since the seminal work, silicone wristbands (WBs) have been increasingly used to facilitate human exposure assessment, as using WBs as a wearable sampler offers new insights into measuring chemical risks involved in many ambient and occupational scenarios. However, the literature lacks a detailed overview regarding methodologies being used; a comprehensive comparison with other approaches of personal exposure assessment is needed as well. Therefore, the aim of this review is fourfold. First, we summarize hitherto conducted research that employed silicone WBs as personal passive samplers. Second, all pre-analytical and analytical steps used to obtain exposure data are discussed. Third, we compare main characteristics of WBs with key features of selected matrices used in exposure assessment, namely urine, blood, hand wipes, active air sampling, and settled dust. Finally, we discuss future needs of research employing silicone WBs. Our work shows a variety of possibilities, advantages, and caveats associated with employment of silicone WBs as personal passive samplers. Although further research is necessary, silicone WBs have already been proven valuable as a tool for longitudinal assessment of personal exposure.

## 1. Introduction

Production, use of, and exposure to chemicals are an inseparable part of technological development [1]. Natural processes, such as forest fires, can also be a source of contaminants [2]. In consequence, humans and wildlife are exposed to a myriad of pollutants that may cause negative health effects [3].

Given the diverse nature of environmental pollution sources, paired with significant knowledge gaps regarding their manner of action when in contact with a human, it is essential to gain details concerning their possible effects on human health. A fundamental step in human health risk assessment is exposure measurement [4]. Therefore, along with the growing number and diversity of synthesized chemicals, the importance of instruments that reliably assess human exposure grows. Only recognized risks can be mitigated through raising awareness and developing informed policies [5]. Although exposure assessment studies appear to be extremely valuable from a scientific point of view, the methods used to quantify exposure vary greatly. Even considering only chemical factors, so far, we do not have universal methods that would enable the assessment of exposure to substances with very diverse physico-chemical properties.

From a practical point of view, we would expect to be able to reliably estimate the average body burden by measuring the concentration of a specific substance or its degradation product/metabolite, preferably using non-invasive sampling methods. Assessment of exposure to environmental pollutants is usually carried out either by performing human biomonitoring (HBM), which is currently considered the gold standard, or by investigating environmental media.

HBM of exposure to chemicals, based on measuring concentration of chemicals in biological matrices, such as urine, blood, or hair, is a frequently used approach [6]. Its main feature is an ability to determine the internal dose of chemicals, regardless of the route of exposure. As a result, it provides the most relevant data for risk assessment, which makes it a powerful [7] and increasingly popular technique in exposure science [8,9].

The concentration of a xenobiotic or its metabolite in the body depends on many factors, including the dose absorbed, the frequency of exposure, and the rate of biotransformation and elimination from the body [10]. For internal dose estimation based on biomarker concentration, knowledge of its pharmaco/toxicokinetics is of fundamental importance [11]. Based on their biological half-life, xenobiotics can be roughly divided into two groups: non-persistent, such as phthalate esters (PEs) and contemporary-use pesticides, which are excreted within several hours from exposure [12]; and persistent organic pollutants (POPs), including polychlorinated biphenyls (PCBs) and dioxins, that have biological half-lives spanning years [13]. For POPs, a single-timepoint measurement in appropriate matrix (typically blood) is sufficient for reliable exposure assessment. Non-persistent chemicals, however, often exhibit high intraindividual variability of biomarkers’ concentration, warranting repeated sampling for accurate exposure estimation. To improve exposure assessment of these chemicals, simultaneous environmental sampling may be conducted [12]. Approaches providing average integrated data over a specified period of time would be particularly useful. Given the transitory nature of non-persistent organic pollutants and the scarcity of information regarding effects of emerging pollutants (both non-persistent and POPs) on human health, there appears to be a dire need for an effective methodology to be developed that would allow for reliable personalized long-term exposure assessment.

Another approach often employed in studies regarding exposure assessment is investigation of environmental media. The range of media used for such research is broad and includes various sampling methods. Environmental media most often analyzed in exposure science are water [14], soil [15], air [16], and dust [17]. Although this approach has a long use history, and throughout the years has provided science with an array of important facts, it is the personal samplers (active air samplers, hand wipes, silicone samplers) that are attracting growing interest among researchers.

Silicone samplers offer a cheap and easily accessible tool for chemically broad environmental sampling, posing as an alternative to expensive active air samplers [18,19]. Although most silicone samplers are used as personal samplers in the form of a wristband (WB) [20], some researchers employed brooches placed on the outer layer of clothing [21], strips [22], or stationary samplers, for example, in indoor [23] or outdoor [24] air monitoring. The building material of said samplers in most cases is poly(dimethylsiloxane) (PDMS), which possesses a set of attributes allowing for its implementation in exposure assessment studies regarding a wide variety of chemicals (see next section).

Considering that most data obtained in exposure assessment studies are made use of in epidemiological research, a quest for the perfect matrix and its sampling method is continuously underway. The purpose of this review is to comprehensively summarize the recent (2014–2021) advances in development of exposure assessment methods that use silicone wristbands as personal passive samplers and to compare silicone wristbands to other approaches in exposure science.

## 2. PDMS as a Sampler Material

PDMS is the most common silicone polymer [25]. Its long history of use in virtually all aspects of analytical chemistry—from sampling to final separation—has been extensively reviewed by Seethapathy and Górecki [26]. PDMS use is so widespread that in many papers, the terms ‘PDMS’ and ‘silicone’ are used interchangeably (e.g., Bergmann et al. [19], Vidi et al. [27], S. Wang et al. [28]), and we follow this pattern throughout our review. One should bear in mind, however, that there are many silicone materials available [29].

The chemical formula of PDMS is (CH_3_)_3_SiO[Si(CH_3_)_2_O]_n_Si(CH_3_)_3_ [26]. The number of monomeric units (n), ranging from just a few to several thousands, strongly affect the mechanical properties of the material. Short-chain PDMS are low-viscosity fluids, whereas the long-chain PDMS form solids [30], albeit an addition of filler (usually SiO_2_) is needed to reinforce the structure [31]. The proportion of the filler in the final material may vary, and it affects not only the mechanical properties, but also the permeability of the material [32].

A raw silicone sampler contains oligomers that will likely interfere during the post-deployment analysis [33,34,35]. Indeed, in a study by Rusina et al. [29], the release of oligomers after exhaustive extraction with ethyl acetate for ten silicone rubbers was tested. In all cases, a substantial loss of mass was observed after the process (2.0–4.2%). Moreover, Anderson et al. [36] and O’Connell et al. [20] showed that improper cleaning procedure leads to high background noise in gas chromatography—mass spectrometry (GC-MS), further emphasizing the role of pre-deployment treatment of silicone samplers; see section “pre-deployment cleanup” for further discussion.

However, PDMS has a number of remarkable features that, taken together, make it an excellent material for a single-phase passive sampler. Due to a flexible backbone and the small size of methyl groups, PDMS exhibits high diffusivity, allowing many different compounds to be sequestrated [26], from air, as recently demonstrated in a series of chamber [37,38], indoor [39,40,41], and field studies (e.g., Bergmann et al. [19], O’Connell et al. [20]). These papers also provide theoretical background, data on PDMS-air partitioning and uptake kinetics of many compounds, and discuss other aspects of passive sampling with wristbands and other PDMS samplers as well. Although PDMS is hydrophobic in nature, it offers significant advantage in sampling moderately polar compounds compared to other popular polymers, such as low-density polyethylene [42]. Finally, silicone exhibits low reactivity [26], is affordable [29], and may be obtained in various shapes and forms, such as sheets, rods, or wristbands.

## 3. Emergence of Silicone Wristbands in Exposure Assessment

**Figure 1:**
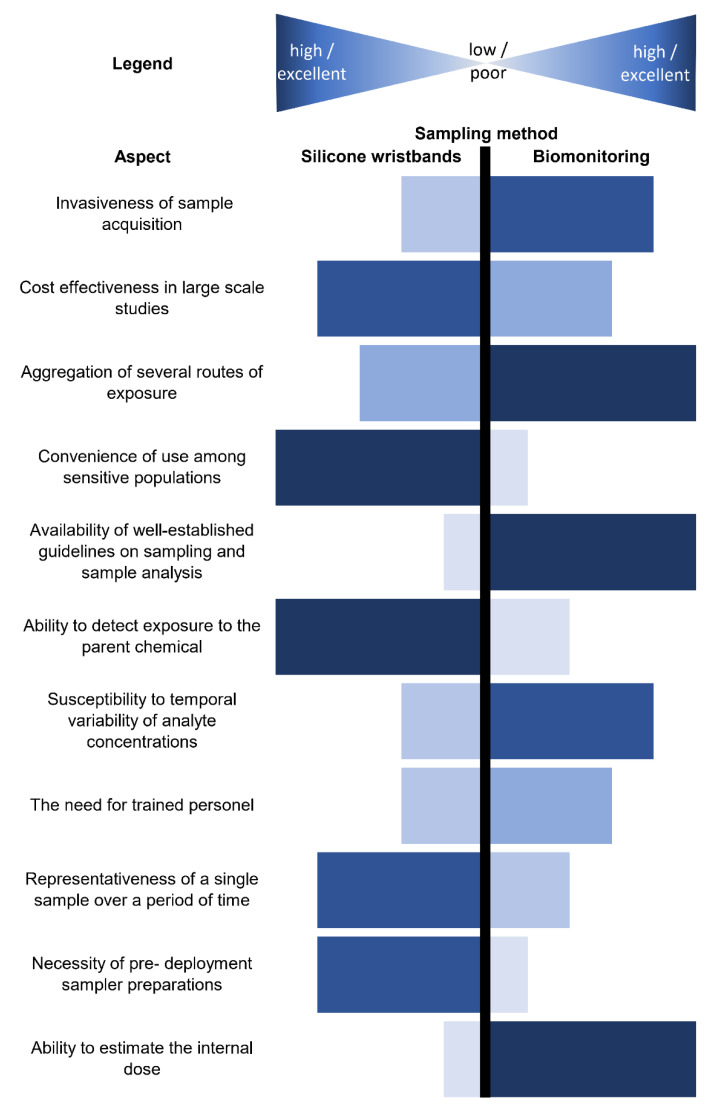
Comparison of attributes of exposure assessment methods with the use of WBs and biomonitoring.

With the plenitude of available sampling methods, one of the emerging devices in the field is a silicone wristband. Popularized as an inexpensive fashion accessory by Lance Armstrong in the mid-2000s [43], it drew scientists’ attention as a passive sampling device nearly a decade later [44]. After the first scientific paper was published [20], many works on this subject have been published in a relatively short period of time. Silicone wristbands are most commonly applied as personal passive samplers in human exposure assessment studies, and as such convey information regarding different routes of human exposure (dermal, inhalatory). Silicone wristbands offer an array of advantages as tools in personal exposure research (Figure 1).

**Figure 2:**
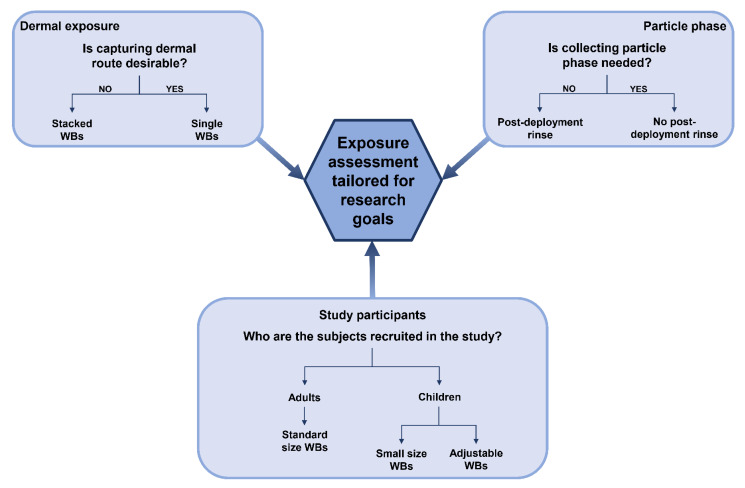
Possibilities of deployment of silicone wristbands (WBs). For references, see section “Emergence of silicone wristbands in exposure assessment”.

The low cost of WB application has a considerable influence on study design, as it allows one to assemble a greater number of study participants without being overly expensive [18,45]. WBs are also non-invasive, which enhances participant compliance [46,47], as the only challenging aspect of the study that the study participants have to withstand is wearing the WBs on their wrists for the duration of sampling period. Small size and unobtrusiveness of these samplers makes this method suitable for application among sensitive populations, like the elderly, children (Figure 2), or pregnant women. The ease of deployment of those samplers also enables the sampling to be carried out by anyone, as it does not require any prior training [45,48].

If the sampler-to-skin contact during the sampling period is not prevented, WBs can provide information about both inhalatory and dermal routes of exposure [21]. This can be considered both an asset as well as a drawback, as it blends two exposure pathways, making it problematic to distinguish a source of a given chemical; however, if desired, WBs can be used as a passive air sampler only [20,49] (Figure 2). WBs also appear to be useful for analysis of metabolites excreted through skin, such as cotinine, a metabolite of nicotine [50]. However, reports of this aspect of their usage are very scarce. Furthermore, when applied as personal samplers, WBs are carried across various microenvironments, so the chemical analysis that follows provides a time-weighted average (TWA) of several exposure episodes taking place over the duration of the experiment [45,51,52]. It is worth noting here that the determination of TWA is possible only in the linear range of uptake of substances from the surrounding environment [53], which is applicable for the semivolatile organic compounds (SVOCs) requiring at least a dozen or so days to achieve equilibrium with the wristband material. In contrast, volatile organic compounds (VOCs) quite quickly reach equilibrium with the wristband material and therefore their content in the band corresponds to the proportional concentration of the substances in the air during the last few hours of exposure [37].

## 4. Search Engine and Exclusion Criteria

The selection of reviewed articles was carried out using PubMed, Web of Science, and Scopus search engines. Upon searching the code-phrase: “silicone wristbands”, the number of publications of interest was 53.

Excluding papers from the initial compilation was consequent to the study’s methodology being described insufficiently in comparison to other research papers. This study focuses on descriptions of original research, which resulted in exclusion of review articles. The main focus of this review is set on application of silicone wristbands as personal passive samplers; therefore, experiments that included different forms of these passive samplers, such as silicone brooches, were excluded, due to consequent differences in monitored routes of human exposure. The cutoff paper publication date for our review was the 31 May 2021. The number of publications of interest post the employment of excluding factors was 45.

The vast majority of reviewed studies was carried out on various populations among inhabitants of the United States of America (>64%); other studies had been done in Europe (The Netherlands, France, Italy, Belgium), Peru, Brasil, Chile, Uruguay, Dominican Republic, Canada, Bangladesh, Senegal, and China (Table 1). Sampling timeframes described in reviewed articles varied from 2012 to 2019, and their duration from 0.3 to 34 days (for human exposure), with one study examining period lasting 161 days (exposure chamber). The median duration of a sampling period was 7 days. The largest study population consisted of 255 participants, and the least numerous had 2. A little over a half of reviewed studies examined exposures using WBs among adults (55.5%), several studies described analysis carried out on a population consisting of children (16.3%), and other explorations had been carried out on groups including both children and adults and/or adolescents. Most research concerned estimating ambient exposure among study participants (79.1%), with occupational-exposure studies being less prolific.

## 5. Chemical Analysis of Silicone Wristbands

Popularity of passive sampling with silicone WBs has increased in recent years, thanks to the seminal paper of O’Connell et al. [20]. Since then, the methodology of application of said wristbands has been evaluated, refined, and repeatedly validated in many studies carried out in diverse settings since 2014, enabling researchers to determine qualitatively and quantitatively the presence of a wide range of substances [19,20,21,79], such as pesticides [24], flame retardants [57,60,62,63], polycyclic aromatic hydrocarbons [18,19], or nicotine [64].

Although the majority of WBs employed in studies conducted since 2014 had been purchased from the same source (www.24hourwristbands.com, accessed on 2 December 2021), the reproducibility of performance of WBs obtained from the same or different sources has not yet been determined. Moreover, accessibility of commercially available WBs, precleaned and ready for application, is poor. These issues are definitely worth solving in the nearest future.

The laboratory procedure regarding handling of wristbands as passive samplers usually consists of several steps. In most cases, WBs require cleaning both prior to and post their deployment. The next phase of sample preparation is extraction, followed by post-extraction sample cleanup. Observed variations in conduction of pre-deployment cleanup, as well as extraction include the use of varying technologies: shakers [20,24], Soxhlet extraction sets [58,72], or vacuum ovens [61,67], as well as diverse amounts of different solvents. The extraction step, although in the technological sense is rather comparable among reviewed studies, varied across the usage of sorbents and elution solvents. A summary of methodology described in reviewed papers can be found in Table 2. Please note that the details included in each row feature a set of information drawn directly from the published paper. 

### 5.1. Pre-Deployment Cleanup

Commercially available wristbands, usually worn as a gadget, may contain numerous impurities from raw materials, but also from their manufacturing, and thus cannot be directly used for sampling. We have not identified a single study that documented qualitatively and quantitatively the contaminants present in commercially available silicone wristbands. Due to this aspect, the bands purchased for research purposes should be properly cleaned before use.

Employment of a uniform washing step for all WBs used in the experiment results in diminished and levelized background noise observed during instrumental analysis, which is reproduced among all used samplers. 

Among reviewed articles, four main approaches regarding pre-deployment cleanup were noted: Soxhlet extraction, performing an agitated wash of WBs, simple rinse or soaking WBs in solvents, and high temperature conditioning.

Most studies opted for a conventional mean of cleaning applied WBs and used Soxhlet extraction for that step. That method, although many up-to-date techniques have come out since its development, has an advantage of being robust and relatively cheap. Duration of Soxhlet extraction varied from 12 h per one cycle (with two cycles conducted) [58,70,72] to up to 3 days (per entire cleaning procedure) [73].

Other approaches substituted Soxhlet extraction with a series of agitated washes of WBs in solvents of different polarities. This technique significantly reduced the time needed to complete the procedure (in comparison to Soxhlet extraction), as the longest reported routine in total took 12.5 h and consisted of five solvent changes (each cycle took 2.5 h) [68]. The cost of applying this technique can vary heavily depending on the amount and purity of solvents used per a number of wristbands or their weight. Agitation of a wash was obtained most commonly via the use of a magnetic plate stirrer [65], an orbital (at the speed of 60–120 rotations per minute) [18,20,71], platform (60 rpm) [68], or overhead (60 rpm) shaker [24], with one study using ultrasonication for that purpose [78]. Performing an agitated wash can be considered more accessible, as it requires the use of common laboratory equipment, unlike Soxhlet extraction.

Some studies performed the cleaning step through washing WBs in varying solvents several times [19,48,52,62], which definitely is the quickest of all described approaches.

Several studies opted for temperature conditioning of WBs as the technique of choice for performing the cleanup step. Conditioning required temperatures up to 300 °C to be achieved and held on for a time in a range of 180 min up to 48 h [36,46,55,61,67,77]. Anderson et al. [36] evaluated this cleanup method by examining the total ion chromatogram, providing pictorial evidence of its efficiency in removing prominent amounts of oligomers.

It is worth noting that one of the aspects of cleanup procedure that requires further investigation is a sufficient solvent volume/weight/number of simultaneously washed WBs ratio. Unfortunately, no study assessed the influence of the WB precleaning procedure on the target analyte uptake, its stability, or its recovery during further extraction. As noted earlier, no identification of manufacturing-related impurities in silicone material used in WBs production has been performed to date.

### 5.2. Post-Deployment Cleanup

During the sampling period, silicone wristbands inevitably come into contact with many materials and chemicals, both environmental (personal care products, dust, food, cleaning products, petrol, oil, and others) and human body-derived (sebum, sweat). In order to tentatively cleanse the surface of the sampler from loosely bound particulates, most of reviewed studies opted for rinsing WBs with the use of deionized water and isopropanol [19,20,48,57,59,69], whereas others opted for the use of methanol in place of isopropanol [18,71]. Finally, in some studies the surface of the sampler was not cleaned after deployment [49]. Overall, descriptions of this step of the analysis usually lack information regarding volume of used solvents or duration of this part of the protocol. Additionally, none of the available studies assessed the cleanup efficiency (e.g., amount of the analyte in rinsing solution and in the silicone matrix). No information was found in any of the publications whether the authors analyzed the rinse wash, which is the generally accepted practice for hair analysis in forensic toxicology [80].

### 5.3. Extraction

The sample extraction step is of utmost importance, as its efficiency, selectivity, and reproducibility will determine the amount of analytes of interest isolated from the processed matrix into the extract. This stage of sample preparation had been carried out in the reviewed research papers by washing post-exposure wristbands in a solvent. Most commonly a cycle (or series of cycles) of agitated WB wash(es) were performed, with the use of either an orbital shaker [18,20,36,45,48,59,67,68,69,75,77], an overhead shaker [24,71], a magnetic stir plate [65], Soxhlet extraction [51], or sonication [21,28,47,58,63,70,72,74,76,78]. The most frequently applied solvent of choice was ethyl acetate [20,24,55]. In the majority of cases, the extraction procedure corresponded a great deal with the pre-exposure WBs cleanup protocol [24,51,68], which is obviously understandable, as the aim of primary WB precleaning, before applying them in a study, is to remove contaminants, including analytes of interest, and therefore attain a blank sampling matrix to be applied in the experiment. Some studies opted for WB fragmentation upon carrying out extraction [51,58,68,70]. Extraction efficiency was evaluated throughout some reviewed studies, starting with O’Connell et al. [20], as their study confirmed the operational efficiency of extraction (90% recovery of the total amount of acenaphthalene-D_8_, fluorene-D_10_, phenanthrene-D_10_, pyrene-D_10_) carried out by their design (via fortification of WBs with standards) that later became a template for other studies regarding this sampling method; the spike test, however, was not done in every study. Variability of analyte levels between fortified WBs that had been evaluated in the same study has also been proven to be very satisfactory (relative standard deviation <13%), therefore validating the capability of silicone WBs to be applied in exposure assessment studies. Surrogate standards, when applied to evaluate extraction efficiency, were added either directly onto the samples before the cleanup [68], or before extraction [46], whereas internal standards were added either before extraction [51,57], or right before analysis, directly into the prepared extract [62].

### 5.4. Post-Extraction Cleanup

Raw extracts attained during sample processing, in order to be useful for a chosen instrumental analysis, tend to be further purified. Among reviewed studies, the most commonly applied approach was solid phase extraction (SPE) [51,77]. This sample preparation step depends crucially on the chemical properties of analytes of interest, as the interactions between the SPE sorbent, eluent, and analysed substances determine the efficiency and selectivity of the process [81]. Most studies that opted for SPE finalized the analysis by the use of gas chromatography–mass spectrometry (GC-MS) [28,47,72]. Performing SPE prior to GC-MS is meant for separating analytes of interest into several distinct fractions, therefore avoiding coelution of substances and mutual interference during analysis. Popular SPE sorbents used among reviewed articles are: C18 [52,61,62], silica gel [58], and Florisil [58,70]. One of the reviewed articles opted for performing post-extraction cleanup (preluding SPE) of WBs via filtration with the use of 0.2 μm PTFE membrane [51] to deprive the extract of larger particles.

### 5.5. Other Methods

It is necessary to take notice to the research papers not listed in Table 2, regarding employment of silicone wristbands as personal passive samplers for analysis of nicotine [50,64], cotinine, and tobacco-specific nitrosamines [50]. Said studies were not included in Table 2 due to significant methodological differences from all the other studies, therefore making it inconvenient to present within our formed outline. Both studies present the use of QuEChERS (Quick, Easy, Cheap, Effective, Rugged, and Safe) extraction technique for nicotine and cotinine analysis. QuEChERS is a quick and cheap method of sample preparation for determination of pollutants residues, e.g., pesticides [82], most common in food analysis. It is a routine dispersive SPE step consisting of single-phase extraction, liquid-liquid partitioning, and addition of salts (e.g., magnesium sulphate, sodium chloride).

## 6. Qualitative and Quantitative Analysis

Silicone WBs have already been shown to be suitable for analysis of a wide array of chemicals. Qualitative methods may include over 1300 analytes [79]. Moreover, a framework for unknown screening using silicone WBs and GC coupled to high-resolution mass spectrometry was recently proposed [83]. Ease of use and capturing capabilities of silicone WB make it an excellent tool for studying exposure to emerging contaminants at a personal level [48].

**Figure 3:**
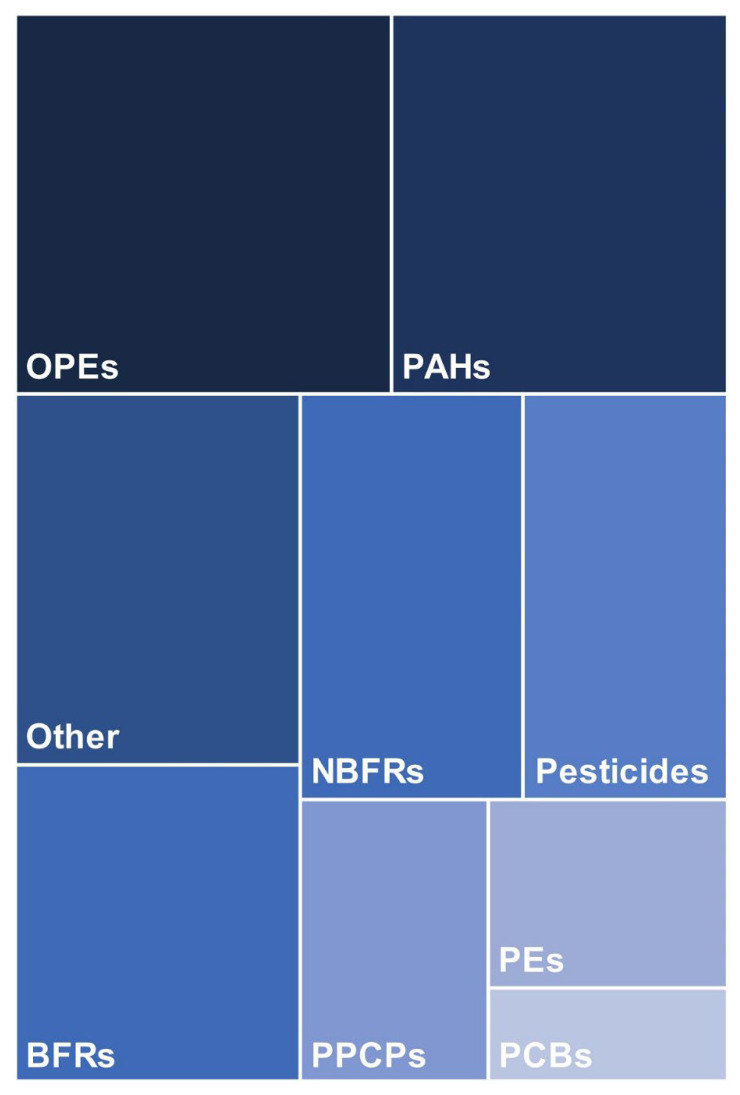
Groups of pollutants analyzed quantitatively in population studies using silicone wristbands. The proportions were computed after assigning a score of 1 to every group per every paper that included quantitative analysis of at least one analyte from the group. Abbreviations: BFRs, brominated flame retardants; NBFRs, novel brominated flame retardants; OPEs, organophosphate esters; PAHs, polycyclic aromatic hydrocarbons; PCBs, polychlorinated biphenyls; PEs, phthalate esters; PPCPs, pharmaceuticals and personal care products.

Quantitative analysis of silicone wristbands also may include many chemicals (Figure 3). For instance, Doherty et al. [46] quantified 199 chemicals from several classes, including pharmaceuticals and personal care products (PPCPs), pesticides, and flame retardants. In this work, compounds with logP values spread throughout over nine orders of magnitude were captured simultaneously. Notably, WBs’ capabilities as a sampler allow the study of ratios between compounds of similar structure, facilitating the identification of exposure source, such as Firemaster 550 in case of OPEs [51] or secondhand tobacco smoke for nicotine and cotinine [50]. The variety of chemicals analyzed in silicone WB is depicted in Figure 3. To date, over 450 different chemicals have been quantified in silicone WBs; the full list is provided in Supporting Information 1 of Supplementary material, Table S1.

However, the use of PDMS as a sorbent material does have its limitations. To our knowledge, no study so far has quantified per- and polyfluoroalkyl substances (PFASs), an important group of emerging pollutants [84], in silicone WBs. Indeed, it has been pointed out that hydrophobic properties of PDMS make it unsuitable for sampling of perfluorooctane sulfonic acid, a well-known PFAS, in water [85]. Extraction efficiency of several other PFASs from water samples using PDMS rods was reported low as well [86]. A similar outcome may be expected for many PFASs sampled in air with a silicone WB [87]. Some (semi)volatile, non-ionic PFASs (e.g., fluorotelomer alcohols) might be an exception. However, to our knowledge, no experimental data on this matter are available to date.

Moreover, discrepancies in presentation of quantitative results exist. Some researchers use analyte mass per entire wristband (e.g., Dixon et al. [48], Xie et al. [78]), whereas others share results as analyte mass per unit mass of the wristband (usually per one gram; e.g., Hammel et al. [72], Wise et al. [74]). These differences may hinder comparisons between the studies [51]. Because wristbands of various sizes are used (e.g., Gibson et al. [62], Quintana et al. [50], Xie et al. [78]), we recommend using analyte mass per unit mass of the wristband as a more versatile approach.

## 7. Comparison of Wristbands with Other Matrices

Since the seminal work by O’Connell et al. [20] was published, several researchers conducted studies involving simultaneous collection of different biological and environmental matrices to gather more exposure data and compare silicone WBs to other means of exposure assessment. Spearman correlation coefficient (r_s_) was used most frequently to determine the strength of association. Although many gaps of knowledge still remain, some remarks can already be made and are provided below.

### 7.1. Biological Matrices

#### 7.1.1. Urine

Urine is an easily accessible biological matrix [88], preferred for most non-persistent chemicals [89] and representing internal exposure level [6]. Therefore, it is not surprising that urine was nearly the only biological matrix WBs were compared to (Supporting Information 2 of Supplementary material, Table S2). WBs correlated moderately well with urine in many, but not all, cases.

Urinary concentrations of 1-hydroxy- metabolites of polycyclic aromatic compounds (PAHs), namely naphthalene, phenanthrene, and pyrene, corresponded well with concentrations of parent compounds in WBs (r_s_ = 0.48–0.76, *p* < 0.05, Table S2). Weaker associations were found comparing these chemicals to their other metabolites or between fluorene and its metabolites [48].

Inconsistent results were obtained in studies investigating silicone WBs–urine relationship while assessing exposure to OPEs. For instance, low and statistically insignificant correlations were observed between triphenyl phosphate (TPHP) in WBs and its metabolite, diphenyl phosphate (DPHP), in urine [49,51,72,78], except for Wise et al. [74] (Table S2). DPHP, however, is not a specific (unique) metabolite of TPHP, so concurrent exposure to other OPEs possibly overshadowed the true link. Complex, route-specific, or unknown metabolism and pharmacokinetics may therefore explain to some extent limited agreement between WBs and urine [51,78]. However, if a parent compound and its specific metabolite were considered, such as tris(1,3-dichloroisopropyl) phosphate and bis(1,3-dichloroisopropyl) phosphate, respectively [90], better correlations between WBs and urine were observed, ranging from 0.43 (*p* < 0.01) [78] to 0.59 (*p* < 0.0001) [51]; however, a trend was only observed in Nguyen et al. (r_s_ = 0.34, *p* = 0.08) [49], and Wise et al. [74] reported a weak and statistically insignificant relationship (r_s_ = 0.24, *p* > 0.05). Tris(1-chloro-2-isopropyl) phosphate (TCIPP) and bis(1-chloro-2-isopropyl) 1-hydroxy-2-propyl phosphate (BCIPHIPP) can also be considered such a pair, with TCIPP being the parent compound detected in WBs, and BCIPHIPP the urinary biomarker [91]. To date, the correlation analyses of these analytes yield contradictory results [49,51,74], despite BCIPHIPP being frequently detected in urine and showing good reproducibility over time [92]. Dietary exposure to certain OPEs, which is not captured by WBs, may also contribute to unsatisfactory correlations with urine [78]. Further research is necessary to elucidate these discrepancies.

In general, results in WBs correlated moderately well with urinary concentrations of PPCPs or their metabolites (Table S2). Nicotine and cotinine in WBs were closely associated with urinary cotinine (r_s_ > 0.84, *p* < 0.01), establishing an exposure-response relationship [50,64]. The strength of observed association and pharmacokinetic data suggest that WBs may have also captured nicotine and cotinine excreted in sweat [50] and thereby partially reflect internal exposure. In a study focused on PPCPs exposure in children [76], PPCP concentrations in WBs were moderately associated with concentrations in urine (r_s_ 0.51–0.66, *p* < 0.0001), except for bisphenol A (BPA) (r_s_ = 0.23, *p* < 0.05). The proposed explanation was that for BPA, in contrast to other PPCPs (e.g., parabens), dietary route is a main source of exposure. In consequence, WBs were not able to capture most of the BPA participants were exposed to. As a similar phenomenon was observed in the case of TPHP [78], an OPE detected in foodstuffs [93,94], it can be speculated that low WBs-urine correlation accompanied by high abundance of metabolite/parent compound in urine implies a dietary pathway as a main source of exposure, whereas high concentrations in both WBs and urine suggest otherwise.

Such approach was used in a study of exposure to phthalate esters (PEs) among nail salon workers [22], where high abundance of di(2-ethylhexyl) terephthalate in WBs and its metabolites in urine confirmed the occupational character of exposure, rather than dietary. This example demonstrates how data obtained with WBs can enrich a biomonitoring study. In turn, Hammel et al. [72] showed weak or moderate correlation (r_s_ 0.3–0.56, *p* < 0.01) between five of seven PEs with paired WBs and urine data (Table S2) among children in an ambient exposure setting.

It should be noted that several factors should be considered when evaluating correlations between these matrices. As noted earlier, silicone WBs offer a wide range of sampling timeframes, ranging from hours [20] to weeks [19] and, possibly, months, depending on study design. In turn, for many chemicals, a single urine sample reflects only recent exposure, within several hours before collection [95,96,97,98,99]. Therefore, continuous, fully adjustable sampling using silicone WBs should be accompanied by parallel urine collection to perform complementary, longitudinal exposure assessment. Some researchers accounted for that by pooling urine samples [51,62,74], but others collected only a single spot sample [48,50], which may have impacted the observed associations. Moreover, urinary flow is known to be variable and influenced by many short-term (e.g., hydration status) and long-term parameters, such as age and BMI [6]. Repeated sampling is known to reduce the effect of short-term variations on the urinary flow rate, therefore improving exposure assessment [89]. Nevertheless, urine is a widely used and acknowledged matrix [6], especially since exposure to nonpersistent chemicals began to attract growing attention [12]. Nearly all nationwide biomonitoring studies include urine collection [100], with the first dating back to 1970s and 1980s [101]. There is also a large body of methodological literature focusing on opportunities and caveats in urine analysis (e.g., Barr et al. [11], Faÿs et al. [102], Franklin et al. [103], Klimowska et al. [104], Meeker et al. [105], Needham et al. [106]). In contrast, WBs have been in use for exposure assessment only since 2014 [20], and no population-scale study has yet been conducted. In addition, although a few methodological papers have already been published [20,36,37,38], many aspects of WBs sampling need to be investigated further (see Section “Future prospects”). Additionally, urine is known to account for all routes of exposure [6], whereas WBs generally capture dermal, inhalatory, but not dietary route [21,22,72,74]. As noted earlier, however, a single WB may cover a much longer period of time than a single urine sample, which is a notable feature in longitudinal studies. Moreover, WBs are far less demanding in terms of transportation and storage conditions [20,36,55]. WBs can be therefore considered a cheaper and less burdensome alternative to urine.

#### 7.1.2. Blood

Only two studies investigated the relationship between pollutants quantified in silicone WBs and in blood [49,58]. In Hammel et al. [58], four out of six brominated flame retardants (BFRs) detected with sufficient frequency in both matrices were moderately correlated (r_s_ = 0.39–0.57, *p* < 0.05) (Table S2). Associations were also observed between congeners within both matrices, identifying PentaBDE commercial mixture as a plausible source of exposure [58]. Furthermore, Nguyen et al. [49] observed a moderate association between decabromobiphenyl ether in plasma and WBs (r_s_ = 0.4, *p* < 0.05). These examples show that silicone WBs may be suitable for estimation of exposure not only to nonpersistent organic pollutants, as discussed earlier, but also to chemicals with long half-lives, such as BFRs [107]. However, further research is necessary to confirm these findings and investigate the WB-blood relationship in other groups of organic pollutants.

### 7.2. Environmental Matrices

#### 7.2.1. Hand Wipes

We touch many objects around us with our hands [108]. Over the past decades, many chemicals have been shown to penetrate the skin barrier effectively, leading to internal exposure (e.g., Appel et al. [109], Lees et al. [110], Piotrowski [111], Weschler et al. [112]). In consequence, monitoring dermal exposure is an important element of thorough exposure assessment [113]. As both hand wipes and WBs may be used for this task, it is tempting to make a comparison between these matrices, which is provided below.

In the majority of cases, a statistically significant positive correlation between individual OPEs concentrations in WBs and in hand wipes was reported (Supporting Information 2 of Supplementary material, Table S3) [51,72]. S. Wang et al. [21] compared hand wipes and wristbands considering OPEs as a group. However, the strength of associations observed in aforementioned studies was weak to moderate, with r_s_ approximately 0.4 between individual OPEs (Table S3).

Levasseur et al. [76] used hand wipes and wristbands as tools for assessment of exposure to phenols in children. The r_s_ values, if calculated, oscillated around 0.5 (Table S3). Detection frequencies of triclosan, methylparaben, ethylparaben, and propylparaben were similar in both matrices, but sharp contrasts were observed for other chemicals, such as BPA (hand wipes and WBs, respectively: 57% vs. 100%) and butylparaben (44% vs. 95%, respectively).

Similar to OPEs, weak to moderate correlations were found between hand wipes and WBs for PEs and their alternatives (r_s_ = 0.24–0.42, *p* < 0.05) [72] (Table S3).

In turn, S. Wang et al. [21] investigated associations between hand wipes and WBs for more lipophilic groups of organic pollutants. Apart from OPEs, PAHs, novel brominated flame retardants (NBFRs), and polybrominated diphenyl ethers (PBDEs) were investigated. Coefficient of determination (r^2^) ranged from 0.58 (PAHs) to 0.73 (PBDEs). Moreover, hand wipes and wristbands showed a very similar profile of captured chemicals.

**Figure 4:**
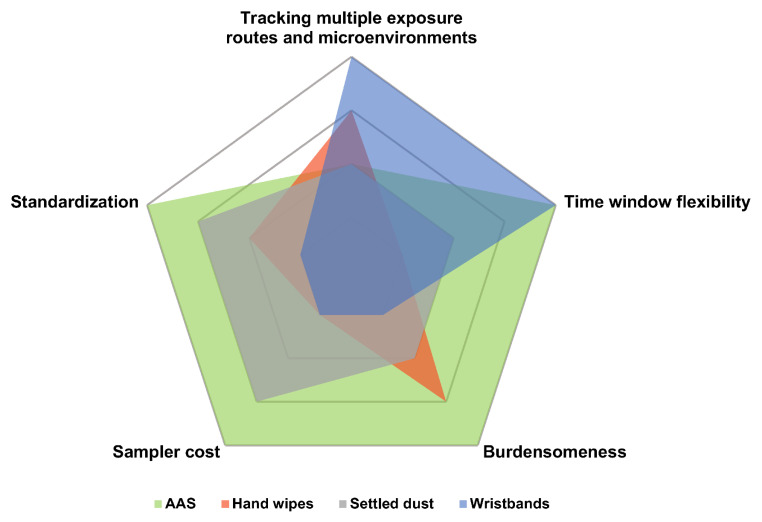
Comparison of key features of wristbands and other environmental media collected during exposure assessment, based on literature review and judgement of the authors. The inner pentagon represents “low/poor” value, whereas the outer one stands for “high/excellent”. For discussion, see section “Comparison with other matrices”. Abbreviations: AAS, active air sampling.

Similarities between the results obtained using WBs and hand wipes are not unexpected, as both matrices are capable of capturing chemicals from several sources—surface contact, vapor phase, and particulates in air [21,22,114] (Figure 4). In both cases, the sampler is small, lightweight, and no power source is needed. Aggregating exposure from several sources, in addition to their low cost [21,115], makes them excellent tools for exposure assessment. Finally, despite long history in exposure assessment [116], the standardization of sample collection of hand wipes also leaves a lot to be desired [117], the key variables being the number of wipes and amount of force applied while wiping the skin [118].

The differences between these matrices, however, are even more striking (Figure 4). Although both matrices capture exposure from similar sources, their main focus appears to be different, with WBs being more effective in sampling vapor and particulate phases, and hand wipes better at reflecting dermal exposure [51]. Furthermore, sampling with hand wipes has been repeatably shown to be susceptible to hand washing, which removes many organic contaminants very effectively and may cause underestimation of exposure [119,120,121]. Due to this fact, participants are asked not to wash their hands for some time prior to sampling, usually an hour [121,122,123], but some sampling protocols require a four-hour interval since the last hand washing [120], which may be considered an inconvenience. In case of WBs, the analytes are absorbed into the polymer, so hand washing should not significantly affect the sampling, although particles on the surface may be removed in the process. Another limitation of hand wipes, partially the consequence of the previous one, is the short time window covered by a single sample and considerable influence of timing of collection [123]. As a result, numerous samples need to be collected in longitudinal exposure assessment. Considering all the characteristics stated above and the fact that concentrations in WBs often correlated better with urine as compared to hand wipes, some authors see WBs as superior to the latter [72,76].

#### 7.2.2. Active Air Sampling (AAS)

AAS is another useful tool in exposure assessment [124,125]. Inhalation pathway appears to be important in exposure to many pollutants [108] that can be monitored by AAS and WBs as well. The comparative discussion below limits AAS to personal sampling.

Dixon et al. [48] analyzed PAHs collected using two devices: an active air sampler (equipped with polyurethane foam (PUF) sorbent and PM_2.5_ filter) and WBs, both being worn simultaneously. A number of detections of each PAH were very similar in WBs and in PUFs, but not in filters, with the notable exceptions of benzo[*b*]fluoranthene, benzo[*k*]fluorantene, benzo[*a*]pyrene, and benzo[*ghi*]perylene, which were detected frequently only in WBs and filters. For PAHs detected in 100% of WBs and PUFs, moderate and strong correlations were observed (r_s_ 0.47–0.71, *p* ≤ 0.03; Table S3), except for pyrene. In turn, S. Wang et al. [21] compared the sum of concentrations of PAHs sequestrated in WBs and an active air sampler connected to a cartridge containing a sandwich PUF-styrene divinylbenzene copolymer (PUF/XAD/PUF), but no associations were found.

S. Wang et al. [21] also compared the total PBDEs, NBFRs, and OPEs quantitated in WBs and an AAS cartridge. No significant associations were found between these matrices for total PBDEs; however, for NBFRs and OPEs, correlations were observed (r^2^ 0.76 and 0.63, respectively; *p* ≤ 0.006).

AAS and WBs share few similarities as personal monitors. Both approaches are capable of precise control of the temporal window covered by an individual sample [125], although AAS is more suitable for short-term studies (typically hours–days) [124,126] (see also next paragraph), whereas WBs, being a passive sampler, is utilized in long-term scenarios (usually days–weeks) [124,126,127]. As AAS samplers and WBs are worn by the subject, both methods are useful in studies involving several microenvironments [51,52,128,129], such as home, office, and vehicle.

In many fundamental aspects, AAS and silicone WBs represent complete opposites (Figure 4). First, in contrast to WBs, AAS requires expensive, heavy, and noisy equipment [125], which may cause discomfort in participants [122], making it impractical for long-term and/or large-scale personal monitoring, especially if several subjects are to be measured simultaneously [124]. Second, AAS by design requires a power source [124] and, due to its technological sophistication [130], researchers’ intervention in case of equipment failure during sample collection [48]. Third, AAS and WBs contrast sharply in the context of standardization. Ever since its first application in personal monitoring [131], AAS was closely linked to occupational exposure assessment [132,133], and numerous manuals, standards, and guidelines were published by reputable sources, such as National Institute for Occupational Safety and Health (e.g., Andrews and O’Connor [134], ASTM International [135]). To our knowledge, no such documents are available for WBs to date. Last but not least, AAS captures only inhalation exposure [124], whereas sampling with WBs includes the dermal pathway as well [21,51]. This aspect was suggested as an explanation of some differences between results obtained with AAS and WBs in both comparative studies [21,48].

#### 7.2.3. Settled Dust

In contrast to the media discussed earlier, quantification of pollutants in settled dust is considered ambient monitoring, rather than personal [136]. Dust is a reservoir of environmental pollutants and may present exposure risk to humans, especially infants and toddlers, due to their mouthing behavior and frequent contact with the floor [137]. In all studies noted below, dust samples were collected indoors with a vacuum cleaner; therefore, the discussion that follows focuses on this method of sampling as well.

Studies assessing OPEs exposure reported few weak correlations between WBs and settled dust, in adults and children alike [72,78]. Additionally, both papers reported that concentrations in WBs better reflected internal exposure (i.e., urinary concentration of biomarkers) than in settled dust.

Concentrations of PEs in settled dust and in WBs corresponded poorly as well [72] (Table S3). Of seven correlations tested, only two weak associations were observed—for diethylphthalate (r_s_ = 0.23, *p* < 0.05) and benzylbutyl phthalate (r_s_ = 0.34, *p* < 0.01).

Modest correlations were found for the majority of PPCPs measured in WBs and settled dust by Levasseur et al. [76]. The lowest r_s_ was reported for butylparaben (0.23, *p* < 0.05), and the highest for triclosan (0.44, *p* < 0.0001) (Table S3). Notably, WBs correlated better with urine than settled dust within every parent compound-metabolite pair, even though study participants were children, who are more exposed to dust than other populations [76].

Some methodological aspects of the aforementioned papers should be noted. All three collected a single dust sample, and only a limited area of each household was vacuumed; this may, to some extent, account for the poor correlations observed [72,76,78]. Moreover, in case of Hammel et al. [72], different instruments were used for quantitation in WBs and settled dust. As noted earlier, two of the papers [72,76] shared the same study population.

From an exposure assessment standpoint, it is difficult to find any similarities between WBs and settled dust (Figure 4). It can be pointed out that settled dust analysis has also been criticized for insufficient standardization [117]. Indeed, many different methodologies are reported for settled dust collection via vacuuming, so even if less popular options such as wiping, brushing, or passive sampling are excluded, substantial variety remains and poses a problem for inter-study comparisons. For instance, sample collection of settled dust can be achieved through simple collection of vacuum cleaner bags from participants or vacuuming the area by researchers using household or specialized vacuum cleaners; each approach collects slightly different material. Moreover, the sample processing, especially sieving, also heavily impacts the results. Diversity of settled dust sampling methods has been reviewed in detail by Mercier et al. [17]. In contrast, a standard reference material of indoor dust (SRM 2585) is available, which facilitates testing and comparing analytical methods between and within laboratories [138]. Moreover, a standard practice for dust collection has been published and is frequently updated [139].

The discrepancy of results described above may result from contrasting features of these matrices (Figure 4). Although the sample collection step is short, settled dust reflects average contamination from a long period of time, even several years [140]. In consequence, the temporal window covered by a settled dust sample may be difficult to control. Questionnaire data (e.g., days since last cleaning, age of a carpet) are used to estimate the time frame [141]. Moreover, humans can be exposed to dust via ingestion, inhalation (finer fractions only), and via direct contact [142], so exposure routes tracked by settled dust and WBs overlap only partially. Finally, settled dust collection via vacuuming requires cumbersome equipment that can be expensive, especially in case of specialized appliances; this poses a problem in large-scale experiments or studies investigating several microenvironments [17].

#### 7.2.4. Other

WBs were also compared to other personal matrices, such as t-shirts [73], silicone brooches [21], or WBs worn on lapels [20,22]. A few studies investigating associations between WBs and various stationary samplers are published as well [24,47,57,143]. However, as such studies are still sparse, the reader is referred to the individual papers.

## 8. Future Prospects

Silicone wristbands are fairly novel sampling tools of emerging applications in exposure assessment studies. Although accessible scientific data confirm suitability of those passive samplers for such research, it should be emphasized that the content of chemicals in wristbands is considered as a semi-quantitative information, as there is no scientific ground for a fully quantitative interpretation. Further refinements and modifications are due in order to standardize methods with their employment. The first aspect of the procedure of wristband use in research that requires unifying, although has been consistent throughout studies mentioned in this review, is construction material of said samplers. Research testing conformance of wristbands coming from several disparate sources should be initiated for further validation of homogeneity and to popularize their employment in different locations around the globe. 

An emergence of commercially available precleaned (and therefore prepared for prompt sampling inauguration) wristbands would be a constructive solution to the aforementioned issue.

Research regarding silicone wristbands should endeavor to achieve uniformity concerning methodology of their use. Accomplishing that will allow for more meticulous and plausible comparison of obtained findings, creating a facility for more comprehensive understanding and assessment of human exposure. 

A possible prospective feature of WBs in exposure assessment studies could be amalgamating this novel sampling technique with geo-tracking of study participants either by a component of a wristband itself, or via the Global Positioning System contained within the vast majority of smartphones. Including any kind of participant trailing system in exposure assessment studies could amount to further cognition of respective environmental contribution to the overall estimated exposure depending on the time spent in each of the surroundings by the study participant, as well as the potential presence of characteristic pollutants that are to be expected in a given setting (workspace, orchard, farmland).

It would also be interesting to investigate associations between WBs and biological matrices other than urine and blood. Hair is arguably the most notable example, as it is also increasingly used in exposure assessment [144] and shares considerable similarities to WBs, such as capturing external exposure [145] and an adjustable temporal window (weeks to months) covered by a single sample [146].

Another opportunity worth considering for future method development is the application of WBs made of materials other than PDMS. Alternative building materials (or their application alongside PDMS in mixed materials passive samplers) that display different properties could potentially allow for broadening the scope of usage of wristbands for exposure assessment, as the methodology might prove to be suitable for employment for sampling further groups of substances displaying miscellaneous chemical attributes. Ionic PFASs may be a prominent example, as their hydrophilic properties prevent efficient sequestration in PDMS samplers.

## Figures and Tables

**Table 1:** The listing of sampling information regarding studies carried out with the use of silicone.

Publication Year	Sampling Year	Country *	Population	Population Age Range (<18 y.o)	*n*	Exposure Setting	Wearing Period [Days]	References
2014	NA	USA	NA	NA	<30	ambient	30	[20] ^†^
2014	NA	USA	NA	NA	8	occupational	0.3, 1.3–1.6	[20] ^†^
2015	2013	USA	adults	NA	50	ambient	7	[54]
2016	2015	USA	adults	NA	40	ambient	5	[51]
2016	2012/2013	USA	children	3–5	92	ambient	7	[52]
2016	2014	SEN	adults, children	NR	35	occupational	5	[55]
2017	2014	PER	adults, children	≥6	68	ambient	30–34	[19]
2017	NR	USA	adults	NA	22	ambient	2	[36]
2017	NR	USA	children	7–9	10	ambient	7	[27]
2017	2012–2013	USA	children	3–5	77	ambient	7	[56]
2018	nd	USA	adults	NA	19	ambient	21	[57]
2018	NR	USA	adults	NA	22	ambient	2	[48]
2018	2016	BEL	adults	NA	30	ambient	5	[24]
2018	2016	USA	adults	NA	30	ambient	7	[58]
2019	2017–2018	USA	adults	NA	101	ambient	7	[21]
2019	2016/2017	USA	adults	NA	10	occupational	0.83–2.08	[22]
2019	2016	BRA	adults	NA	2	ambient	3	[59]
2019	2016	USA	adults	NA	10	ambient	7	[60] ^†^
2019	2017	USA	adults	NA	22	ambient	7	[60] ^†^
2019	2016	USA	adolescents	14–16	97	ambient	7	[61]
2019	2008–?	USA	child-mother pairs	3–5	32	ambient	7	[62]
2019	NR	USA	adults	NA	10	ambient	7	[63]
2019	2017	USA	children	4–14	31	ambient	7,2	[64]
2019	NA	CAN, NED	NA	NA	NA	exposure chamber	1, 4, 10, 30, 50, 71, 91, 161	[38]
2019	NA	USA	NA	NA	NA	NA	7	[65]
2019	NR	NR	NR	NA	10	NR	7	[66]
2019	2016–2017	CHL	NR	NA	27	ambient	5	[45]
2019	NR	NR	NR	NA	16	ambient	18	[67]
2020	2018	URY	children	6–7.8	24	ambient	7	[68]
2020	2019	JPN	adults	NA	5	ambient	5	[69]
2020	2017	USA	adults	NA	72	occupational	1	[18]
2020	2019	USA	adults	NA	88	ambient	5	[70]
2020	2017–2018	USA	adults	NA	101	ambient	7	[47]
2020	2019	DOM	adults	NA	15	occupational	1	[71]
2020	2017–2018	USA	adults	NA	255	ambient	7	[46] ^†^
2020	2017–2018	USA	adults	NA	20	ambient	7	[46] ^†^
2020	2015/2016	USA	children	3–6	77	ambient	7	[72]
2020	2017–2018	USA	children	3–14	53	ambient	7, 2	[50]
2020	2018–2019	FRA	adults	NA	40	ambient	5	[28] ^†^
2020	2018–2019	ITA	adults	NA	31	ambient	5	[28] ^†^
2020	2018	BGD	adolescents/adults	≥14	15	occupational	1	[73]
2020	2018	USA	adults	NA	30	ambient	5	[74]
2020	2018	USA	adults	NA	17	occupational	1	[75]
2020	2017	CAN	adults	NA	45	occupational	0.3	[49]
2021	2014–2016	USA	children	3–6	27	ambient	7	[76]
2021	2018–2019	USA	children	10–17	163	ambient	7	[77]
2021	2018–2019	CHN	Child-mother pairs	≤7	47	ambient	14	[78]

Note: WBs: n—number of tested samples/participants (NR—not reported, NA—not applicable), *—in accordance with ISO 3166. ^†^—studies described within the same paper, individual tested groups separated in this chart due to reciprocal differentiation in presented variables.

**Table 2:** Methodologies applied in reviewed articles (NR—not reported, Y—substances included in the study, N—substances not included in the study).

	Pre-Deployment		Post-Deployment		Extraction		Post-Extraction Sample Cleanup		Analyzed Substances									Instrumental Analysis	
Publication Year	Mechanism	Protocol	Mechanism	Protocol	Mechanism	Protocol	Instrumentation	Protocol	NBRFs	OPEs	PAHs	BFRs	PCBs	PEs	Pesticides	PPCPs	Other		Ref.
2014	Agitated wash (orbital shaker)	3 × EtAc:n-hex (2.5 h), 60 rpm 2 × EtAc:MeOH (2.5 h), 60 rpm	Rinse	2 × DI water 1 × IPA	Agitated wash (orbital shaker)	2 × EtAc,100 mL, (2 h), 60 rpm	NR	NR	N	Y	Y	N	Y	Y	Y	Y	Y	GC-MS	[20]
2016	Thermal conditioning	280–300 °C (48 h)	Rinse	1 × DI water 1 × IPA	NR	2 × EtAc, 100 mL	NR	NR	N	N	N	N	N	N	Y	N	N	GC-ECD	[55]
2016	Soxhlet extraction	1 × EtAc:n-hex, (12 h) 1 × EtAc:MeOH, (12 h)	NR	NR	Soxhlet extraction	1 × n-hex:acetone, (12 h)	Syringe filter (0.2 µm PTFE) SPE cartridges (Florisil, 500 mg)	Filtration Elution:	N	Y	N	N	N	N	N	N	N	GC-MS	[51]
								F1:n-hex (10 mL)											
								F2:EtAc (10 mL)											
2017	Wash	3 × EtAc:n-hex 2 × EtAc:MeOH	Rinse	2 × DI water 1 × IPA	Wash	1 × EtAc, 100 mL, (12 h) 1 × EtAc, 100 mL, (2 h)	NR	NR	Y	Y	Y	Y	Y	Y	Y	Y	Y	GC-ECD, GC-MS	[19]
2017	Conditioning (vacuum oven)	300 °C, 180 min, 0.1 Torr	Rinse	2 × DI water 1 × IPA	Agitated wash (orbital shaker)	2 × EtAc, 100 mL	NR	NR	N	Y	Y	Y	Y	N	Y	Y	Y	GC-MS, GC-MS/MS, GC-µECD	[36]
2017	Soak	EtAc, n-hex, MeOH	Rinse	2 × water 1 × IPA	NR	2 × EtAc, 100 mL	SPE cartridges (C18, 500 mg)	Elution: ACN	Y	Y	N	Y	N	N	N	N	N	GC-MS	[52]
2018	NR	NR	Rinse	1 × DI water 1 × IPA	Dialysis	2 × EtAc	NR	NR	N	N	Y	N	N	N	N	N	N	GC-MS/MS	[57]
2018	Solvent exchange	3 × EtAc:n-hex 2 × EtAc:MeOH	Rinse	2 × DI water 1 × IPA	Agitated wash (orbital shaker)	2 × EtAc 100 mL, 60 rpm	NR	NR	N	N	Y	N	N	N	N	N	N	GC-MS/MS	[48]
2018	Agitated wash (overhead shaker)	1 × EtAc:n-hex, (30 min) 1 × EtAc:MeOH, (30 min):	NR	NR	Agitated wash (overhead shaker)	2 × EtAc, 40 mL, (30 min)	NR	NR	N	N	N	N	N	N	Y	N	N	LC-MS	[24]
2018	NR	NR	Rinse	2 × DI water 1 × IPA	Wash	1 × EtAc 100 mL, (12 h) 1 × EtAc, 100 mL, (2 h)	NR	NR	N	N	N	N	N	N	Y	N	N	GC-µECD	[27]
2018	Soxhlet extraction	1 × EtAc:n-hex, (12 h) 1 × EtAc:MeOH, (12 h)	NR	NR	Sonication	3 × n-hex:acetone, 10 mL	Custom SPE: Florisil (500 mg) and silica gel (12 g; F1 only)	Elution (Florisil):	Y	N	N	Y	N	N	N	N	N	GC-MS	[58]
								F1:n-hex											
								F2:EtAc											
								Elution (silica gel): F3:DCM:n-hex											
2019	Rinse, conditioning	Water rinse, thermal conditioning	Rinse	1 × DI water 1 × IPA	Agitated wash (orbital shaker)	2 × EtAc, 100 mL, (2 h)	NR	NR	NR	NR	NR	NR	NR	NR	NR	NR	NR	GC-MS	[67]
2019	Soxhlet extraction	1 × EtAc:n-hex, (24 h) 1 × EtAc:MeOH, (24 h)	NR	NR	Sonication	2 × n-hex:acetone, 30 mL, (2 h)	Custom SPE (neutral alumina, neutral silica gel, sulfuric acid- silica gel, sodium sulfate)	Elution: DCM (40 mL)	Y	Y	N	Y	N	N	N	N	Y	GC-MS	[21]
							Custom SPE (neutral alumina, neutral silica, Florisil, sodium sulfate)	Elution:											
								F1:DCM (40 mL)											
								F2:EtAc (40 mL)											
2019	NR	NR	Rinse	2 × DI water 1 × IPA	Agitated wash (orbital shaker)	2 × EtAc, 100 mL, 60 rpm	NR	NR	N	Y	Y	N	N	Y	Y	Y	Y	GC-MS	[59]
2019	Thermal conditioning (vacuum oven)	300 °C, (180 min), 0.1 Torr	Rinse	1 × DI water 1 × IPA	NR	2 × EtAc, 100 mL	SPE (C18, silica)	Elution: ACN	Y	Y	Y	Y	Y	Y	Y	Y	Y	GC-µECD, GC-MS	[61]
2019	Soak	EtAc, n-hex, MeOH	NR	NR	NR	2 × EtAc, 100 mL	SPE cartridges (C18, 500 mg)	Elution: ACN	N	Y	N	N	N	N	N	N	N	GC-MS	[62]
2019	Soxhlet extraction		Agitated wash	1 × DI water	Sonication	1 × Acetone:n-hex, 20 mL, (2 h)	Custom SPE (neutral alumina, neutral silica, Florisil, anhydrous sodium sulfate)	Elution:	Y	Y	Y	Y	N	N	N	N	Y	GC-MS	[63]
								F1:DCM											
								F2:EtAc											
			Rinse	1 × IPA			Custom SPE, (neutral alumina, neutral silica, acidic silica, anhydrous sodium sulfate)	elution: F3: DCM											
2019	NR	NR	Rinse	2 × DI water 1 × IPA	Agitated wash (orbital shaker)	2 × EtAc, 100 mL, (2 h), 60 rpm	-	-	N	N	Y	N	N	Y	Y	N	Y	GC-GC/ToF-MS	[45]
2020	Soxhlet extraction	1 × EtAc (3 days)	-	-	Agitated wash (Wrist Action Shaker)	1 × ACN, 30 mL	Syringe filter (0.2 µm, Teflon)	Filtration	Y	Y	N	Y	N	N	N	N	Y	GC-MS	[49]
2020	Agitated wash (platform shaker)	3 × EtAc:n-hex, (2.5 h) 2 × EtAc:MeOH, (2.5 h), 60 rpm	NR	NR	Agitated wash (orbital shaker)	2 × EtAc, 25 mL, (2 h), 60 rpm	SPE cartridges (C18, 500 mg)	Elution: ACN	Y	Y	N	Y	Y	N	Y	N	Y	GC-MS	[68]
2020	NR	NR	Rinse	1 × DI water 1 × IPA	Agitated wash (orbital shaker)	2 × EtAc, 25 mL, (24 h)	SPE cartridges (C18, 500 mg)	Elution: n-hex: DCM (4 mL)	N	N	Y	N	N	N	N	Y	N	GC-MS	[69]
2020	Agitated wash (orbital shaker)	1 × MeOH (10 min) 3 × n-hex:EtAc (1 h), 2 × MeOH:EtAc	Rinse	1 × MeOH	agitated wash (orbital shaker)	2 × 30 mL EtAc, 30 mL, (1 h)	NR	NR	N	N	Y	N	N	N	N	N	N	GC-MS	[18]
2020	Soxhlet extraction	1 × EtAc:n-hex, (12 h) 1 × EtAc:MeOH, (12 h)	NR	NR	Sonication	3 × n-hex: DCM, 10 mL, (15 min)	SPE (Florisil, 8 g)	Elution:	N	Y	N	N	N	N	N	N	N	GC-MS/MS	[70]
								F1:n-hex											
								F2:EtAc											
2020	Soxhlet extraction	1 × EtAc:n-hex, (24 h) 1 × EtAc:MeOH, (24 h)	NR	NR	Sonication	2 × n-hex:acetone, 30 mL, (2 h)	Custom SPE (neutral alumina, neutral silica, Florisil, sodium sulfate)	Elution: DCM	N	N	Y	N	N	N	N	N	N	GC-MS	[47]
2020	Agitated wash (orbital shaker)	2 × MeOH, (10 min), 120 rpm 2 × (1 h): n-hex:EtAc, (1 h), 120 rpm 2 × MeOH:EtAc, 120 rpm	Rinse	1 × MeOH	Agitated wash (overhead shaker)	2 × EtAc, 30 mL	NR	NR	N	N	Y	N	N	N	N	N	N	GC-MS	[71]
2020	Conditioning (vacuum oven)	300 °C, (12 h), 0.1 Torr	Rinse	2 × DI water 1 × IPA	NR	2 × EtAc, 50 mL	SPE cartridges (C18)	Eluted: ACN	N	Y	Y	Y	Y	Y	Y	Y	Y	GC-MS	[46]
2020	Soxhlet extraction	1 × EtAc:n-hex, (12 h) 1 × EtAc:MeOH, (12 h)	NR	NR	Sonication	3 × n-hex:DCM, 10 mL)	SPE cartridges (Florisil, 500 mg)	Elution:	N	Y	N	N	N	Y	N	N	Y	GC-MS	[72]
								F1: n-hex											
								F2: EtAc											
								F3: MeOH											
2020	NR	NR	Rinse	DI water	Sonication	2 × n-hex: acetone, 30 mL, (2 h)	Chromatography column (neutral alumina, neutral silica gel, sulfuric acid-silica gel, sodium sulfate)	Elution: DCM	Y	Y	Y	Y	N	N	N	N	N	GC-MS	[28]
							Chromatography column (neutral alumina, neutral silica gel, Florisil, sodium sulfate)	Elution:F1:DCM											
								F2:EtAc											
2020	Soxhlet extraction	1 × pentane (3 days)	-	-	Agitated wash	ACN	SPE cartridge (Florisil, 500 mg)	Elution: EtAc	Y	Y	N	Y	N	N	N	N	Y	GC-MS	[73]
2020	Agitated wash (magnetic stir plate)	3 × EtAc:n-hex, (30 min), 60 rpm 2 × EtAc:MeOH, (30 min), 60 rpm	NR	NR	Agitated wash (magnetic stir plate)	ACN:MeOH, 20 mL, (1 h), 60 rpm	NR	NR	N	N	N	N	N	N	N	N	Y	HPLC	[65]
2020	NR	NR	NR	NR	Sonication	3 × n-hex:DCM, 10 mL	SPE (Florisil, 8 g)	Elution:	Y	Y	N	Y	Y	Y	Y	N	Y	GC-MS, GC-MS/MS	[74]
								F1: n-hex,											
								F2: EtAc,											
								F3: MeOH											
2020	Agitated wash (orbital shaker)	1 × MeOH (10 min), 120 rpm 2 × EtAc:n-hex (1 h), 120 rpm 2 × EtAc:MeOH (1 h), 120 rpm	NR	NR	Agitated wash (orbital shaker)	2 × EtAc, 30 mL, (1 h), 120 rpm	NR	NR	N	N	Y	N	N	N	N	N	N	GC-MS	[75]
2021	Soxhlet extraction	1 × EtAc:n-hex (12 h) 1 × EtAc:MeOH (12 h)	NR	NR	Sonication	3 × DCM:n-hex	SPE cartridges (Florisil, 500 mg)	Elution:	N	N	N	N	N	N	N	Y	Y	LC-MS	[76]
								F1											
								F2: EtAc											
								F3											
2021	Rinse, conditioning	DI water, 300 °C (180 min)	rinse	1 × DI water 1 × IPA	Agitated wash (orbital shaker)	2 × EtAc	SPE (C18, silica)	Elution: ACN	N	N	N	N	N	N	Y	N	N	GC-ECD, GC-MS	[77]
2021	Sonication	3 × DCM:n-hex, (20 min)	NR	NR	Sonication	2 × DCM: n-hex, 15 mL, (20 min)	SPE cartridges (Florisil, 2 g)	Elution: 1 × n-hex 1 × EtAc	N	Y	N	N	N	N	N	N	N	LC-MS	[78]

Abbreviations: ACN, acetonitrile; BFRs, brominated flame retardants; DCM, dichloromethane; DI, deionized; EtAc, ethyl acetate; F1, F2, F3, numeration of fractions eluted (in accordance to their order of elution); IPA, isopropyl alcohol; MeOH, methanol; NBFRs, novel brominated flame retardants; n-hex, n-hexane; OPEs, organophosphate esters; PAHs, polycyclic aromatic hydrocarbons; PCBs, polychlorinated biphenyls; PEs, phthalate esters; PPCPs, pharmaceuticals and personal care products; SPE, solid phase extraction.

## Supplementary Materials

The following supporting information can be downloaded at: https://www.mdpi.com/article/10.3390/ijerph19041935/s1. Table S1: List of compounds quantified in silicone wristbands in human exposure studies; Table S2: Correlations between chemicals quantified in silicone wristbands and their respective biomarkers quantified in biological matrices; Table S3: Correlations between silicone wristbands and other environmental matrices.

## Abbreviations

AAS: active air sampling; BCIPHIPP, bis(1-chloro-2-isopropyl) 1-hydroxy-2-propyl phosphate; BPA, bisphenol A; BFRs, brominated flame retardants; DPHP, diphenyl phosphate; FRs, flame retardants; GC-MS, gas chromatography-mass spectrometry; HBM, human biomonitoring; NBFRs, novel brominated flame retardants; OPEs, organophosphate esters; PAHs, polycyclic aromatic hydrocarbons; PBDEs, polybrominated diphenyl ethers; PCBs, polychlorinated biphenyls; PBFRs, polybrominated flame retardants; PDMS, poly(dimethylsiloxane); PEs, phthalate esters; PFASs, per- and polyfluoroalkyl substances; POPs, persistent organic pollutants; PPCPs, pharmaceuticals and personal care products; SPE, solid phase extraction; SVOCs, semivolatile organic compounds; PUF, polyurethane foam; QuEChERS, quick, easy, cheap, effective, rugged, and safe; TCIPP, tris(1-chloro-2-isopropyl) phosphate; TPHP, triphenyl phosphate; TWA, time-weighted average; VOCs, volatile organic compounds; WB, wristband.
